# Capnography monitoring during procedural sedation and analgesia: a systematic review protocol

**DOI:** 10.1186/s13643-015-0085-4

**Published:** 2015-07-14

**Authors:** Aaron Conway, Clint Douglas, Joanna Sutherland

**Affiliations:** Institute of Health & Biomedical Innovation, Queensland University Technology, Kelvin Grove Campus, Kelvin Grove, Queensland 4059 Australia; Cardiac Catheter Theatres, The Wesley Hospital, Auchenflower, QLD Australia; School of Nursing, Institute of Health & Biomedical Innovation, Queensland University Technology, Kelvin Grove Campus, Kelvin Grove, 4059 Queensland Australia; Coffs Harbour Health Campus, New South Wales, Australia; Rural Clinical School, University of New South Wales, New South Wales, Australia

**Keywords:** Procedural sedation and analgesia, Conscious sedation, Deep sedation, Capnography, End-tidal carbon dioxide

## Abstract

**Background:**

An important potential clinical benefit of using capnography monitoring during procedural sedation and analgesia (PSA) is that this technology could improve patient safety by reducing serious sedation-related adverse events, such as death or permanent neurological disability, which are caused by inadequate oxygenation. The hypothesis is that earlier identification of respiratory depression using capnography leads to a change in clinical management that prevents hypoxaemia. As inadequate oxygenation/ventilation is the most common reason for injury associated with PSA, reducing episodes of hypoxaemia would indicate that using capnography would be safer than relying on standard monitoring alone.

**Methods/design:**

The primary objective of this review is to determine whether using capnography during PSA in the hospital setting improves patient safety by reducing the risk of hypoxaemia (defined as an arterial partial pressure of oxygen below 60 mmHg or percentage of haemoglobin that is saturated with oxygen [SpO^2^] less than 90 %). A secondary objective of this review is to determine whether changes in the clinical management of sedated patients are the mediating factor for any observed impact of capnography monitoring on the rate of hypoxaemia. The potential adverse effect of capnography monitoring that will be examined in this review is the rate of inadequate sedation. Electronic databases will be searched for parallel, crossover and cluster randomised controlled trials comparing the use of capnography with standard monitoring alone during PSA that is administered in the hospital setting. Studies that included patients who received general or regional anaesthesia will be excluded from the review. Non-randomised studies will be excluded. Screening, study selection and data extraction will be performed by two reviewers. The Cochrane risk of bias tool will be used to assign a judgment about the degree of risk. Meta-analyses will be performed if suitable.

**Discussion:**

This review will synthesise the evidence on an important potential clinical benefit of capnography monitoring during PSA within hospital settings.

**Systematic review registration:**

PROSPERO CRD42015023740

**Electronic supplementary material:**

The online version of this article (doi:10.1186/s13643-015-0085-4) contains supplementary material, which is available to authorized users.

## Background

### Description of the condition

Procedural sedation and analgesia (PSA) is used to control pain and distress during diagnostic and interventional medical procedures. PSA is generally considered to include the stages of moderate and deep sedation of the continuum of anaesthesia [1]. Loss of consciousness is not an intended outcome, and cardiac and respiratory function may not be impaired with the doses and types of sedative and analgesic medications used [1]. However, PSA is still associated with at the least the same or potentially even greater risk of serious sedation-related adverse events, such as death or permanent neurological disability, compared with general anaesthesia [2]. Due to the potential that respiratory function may become impaired as a result of sedation-induced depression of the central nervous system, frequent monitoring is recommended [1, 3]. Respiratory function is usually evaluated by observation of qualitative clinical signs (respiratory rate, depth and effort) and oxygen saturation monitoring [3]. Capnography is a respiratory monitoring device that has become an accepted standard of care for PSA in many circumstances. For example, the American Society of Anesthesiology standards for Basic Anesthetic Monitoring require the use of capnography for both moderate and deep sedation. In the UK, the Academy of Medical Royal Colleges Standards and Guidance for Safe Sedation Practice for Healthcare Procedures also include capnography as a developmental standard for patients receiving sedation where it has not already been implemented into practice.

### Description of the intervention

Capnography monitors use infrared spectroscopy to measure the amount of carbon dioxide in exhaled breath [4]. End-tidal carbon dioxide (ETCO_2_) is the partial pressure of carbon dioxide measured at the end of exhalation, which represents alveolar carbon dioxide concentration [5]. Assuming that ventilation and perfusion is normal, ETCO_2_ is approximately equal to the arterial partial pressure of carbon dioxide [5]. A waveform tracking the level of carbon dioxide is displayed to show changes in carbon dioxide concentration during the respiratory cycle. Changes in ETCO_2_ values and/or the shape of the capnography waveform may aid the diagnosis of iatrogenesis (e.g. ETT malplacement), physiological abnormality (e.g. hyperventilation) or disease states [6].

### Why is it important to do this review?

Hypoventilation or apnoea will theoretically precede hypoxaemia if it is caused by sedation-induced respiratory depression. For this reason, alterations in capnography waveforms and ETCO_2_ levels that indicate hypoventilation or apnoea should be early markers of sedation-induced respiratory depression. Clinical studies provide some evidence to support this hypothesis. A meta-analysis revealed that respiratory depression was 17.6 times more likely to be detected among sedated patients who were monitored with capnography than with standard monitoring alone [7]. However, sedation-induced respiratory depression can be transient, and not all episodes of respiratory depression result in patient harm [8]. In this context, it is important to note that intervening at the onset of an episode of respiratory depression that would have resolved spontaneously without harming the patient may be counterproductive because it could result in inadequate sedation [9]. Rigorous evaluation of the potential clinical benefits, including comprehensive syntheses of the available evidence, is therefore required.

There are few systematic reviews available to inform clinical practice and research regarding how to optimise the clinical application of capnography during PSA. A meta-analysis has been conducted to determine whether capnography detected more respiratory complications than standard monitoring alone [7]. Although it was found in this systematic review that respiratory depression was more likely to be detected when capnography was used during PSA, the clinical benefits arising from increased detection were not examined [7]. A protocol for a Cochrane systematic review that focuses on capnography during PSA has also been published [10]. However, this review will only include studies conducted in the emergency department [10]. We searched the systematic review database, PROSPERO, on 27th April, 2014, with the terms capnography, end-tidal carbon dioxide and sedation. No other published or current systematic reviews were identified that focused on the use of capnography during PSA.

One important potential clinical benefit of using capnography monitoring during PSA is that this technology could improve patient safety by reducing short-term serious sedation-related adverse events, such as death or permanent neurological disability, which are caused by inadequate oxygenation. The hypothesis is that earlier identification of respiratory depression using capnography leads to a change in clinical management that prevents hypoxaemia. As inadequate oxygenation/ventilation is the most common reason for injury associated with PSA, reducing episodes of hypoxaemia would indicate that using capnography would be safer than relying on standard monitoring alone [11].

### Objectives

The primary objective of this review is to determine whether using capnography during PSA in the hospital setting improves patient safety by reducing the risk of hypoxaemia (defined as an arterial partial pressure of oxygen below 60 mmHg or percentage of haemoglobin that is saturated with oxygen [SpO^2^] less than 90 %). A secondary objective of this review is to determine whether changes in the clinical management of sedated patients are the mediating factor for any observed impact of capnography monitoring on the rate of hypoxaemia. The potential adverse effect of capnography monitoring that will be examined in this review is the rate of inadequate sedation.

## Methods/design

A systematic review and meta-analyses adhering to the protocol outlined below will be conducted. Differences between this protocol and the review will be reported.

### Criteria for considering studies for this review

#### Types of studies

Parallel, crossover and cluster randomised controlled trials will be included. Studies that tested non-inferiority or equivalence hypotheses will not be included because capnography monitoring is not used as a direct alternative for standard monitoring. Non-randomised or quasi-randomised trials will not be included.

#### Types of clinical settings

Studies conducted in any hospital setting will be included. Examples of hospital settings include emergency departments, medical-imaging departments, endoscopy suites and cardiac catheterisation laboratories. Dental surgeries/clinics will be excluded due to the potentially confounding influence of different staffing profiles (i.e. the presence of a medical doctor versus a dentist).

#### Types of participants

Studies that included patients (adults or children) who received procedural sedation and analgesia (defined as administration of any intravenous sedative and/or analgesic medications with or without local anaesthesia) will be included in the review. Studies that included patients who received general or regional anaesthesia will be excluded from the review.

#### Types of intervention

PSA was managed by a strategy where capnography monitoring was used.

#### Types of comparison

PSA was managed without capnography monitoring.

####  Types of outcome measures

Primary outcome:

- Hypoxaemia (arterial partial pressure of oxygen below 60 mmHg or percentage of haemoglobin that is saturated with oxygen [SpO^2^] less than 90 % for any period of time). The definition for hypoxaemia used in each included study will be extracted, as it is possible that alternative definitions were used.

Secondary outcomes:

- Change in clinical management

- o change in supplemental oxygen
- o airway intervention (specific interventions used will be extracted)
- o medication titration (as a dichotomous variable)
- o sedation reversal
- o amount of sedative and analgesic medications used (as a continuous variable with a separate analysis undertaken for each medication)

- Unable to complete procedure as it was planned due to inadequate sedation
- Sedation-related adverse events (death, neurological deficits, unplanned conversion to general anaesthesia/endotracheal intubation, unplanned admission to intensive care unit)

As we hypothesise that changing clinicians’ clinical management decision-making is the moderating mechanism for clinical benefits associated with capnography monitoring during PSA, secondary outcomes will be the amount of sedative and analgesic medication used and a composite of whether there was a change in the use of supplemental oxygen, use of an airway intervention or administration of sedation reversal. We also hypothesise that intervening at the onset of an episode of respiratory depression that would have resolved spontaneously may be counterproductive because it could result in inadequate sedation. For this reason, we will investigate the number of procedures needing to be abandoned due to inadequate sedation as a secondary outcome.

### Search methods for identification of studies

Published, unpublished and ongoing studies will be identified by searching the following databases:

- The Cochrane Central Register of Controlled Trials (CENTRAL) (1999 (established) to present);
- MEDLINE (OvidSP) (1966 to present);
- CINAHL (EBSCOhost) (1982 to present);
- ClinicalTrials.gov; and
- World Health Organization International Clinical Trials Registry Platform.

Guidance from the Cochrane Handbook for Systematic Reviews of Interventions, chapter 6.4, was used to formulate the search strategy (Appendices 1, 2 and 3) [12]. Language restrictions will not be imposed. If the study has been considered as potentially eligible based on the abstract, attempts to either have the article translated or have the required data extracted will be made.

### Data collection and analysis

#### Selection of studies

 measuresTitle and abstract screening will be performed by two independent reviewers (AC and CD). Full-text copies of all studies that appear to meet the inclusion criteria will be obtained for further assessment. Second screening will be performed by two independent reviewers (AC and CD) applying all inclusion and exclusion criteria (Additional file 1). Disagreement on eligibility will be resolved by discussion.

#### Data extraction and management

AC and CD will independently extract data using the data extraction form (Additional file 1). Differences of opinion will be reconciled by mutual agreement. Data will be entered into a database (Review Manager (RevMan), version 5) for statistical analysis.

#### Assessment of risk of bias in included studies

AC and CD will undertake the risk of bias assessment of the included studies independently, as guided by the Cochrane Handbook for Systematic Reviews of Interventions [12]. The Cochrane risk of bias tool will be used to assign a judgment about the degree of risk (low risk of bias, high risk of bias and unclear risk of bias) (Additional file 1) [12].

#### Measures of treatment effect

When the measure of the outcome is sufficiently consistent across trials, we will use risk ratios for dichotomous data and mean difference for continuous data with corresponding 95 % CIs.

#### Unit/scale of analysis issues

The unit of analysis is based on the individual patient (the unit that was randomised for comparison of interventions). We will use pre-crossover data for included trials that used a crossover design. As per Cochrane guidance, we will first assess whether data from cluster randomised trials were analysed in a way that accounted for clustering [13]. If not, trial results will be adjusted for inclusion in a meta-analysis. Either an effective sample size will be calculated using the design effect to adjust the study data or the standard error of the estimated intervention effect will be inflated by multiplying the standard error by the square root of the design effect.

#### Dealing with missing data from individuals

As the outcomes selected for this review are measured during the procedure, it is unlikely that there will be missing data. However, a potential reason for missing data could be malfunction of monitoring equipment resulting in a loss of data about intra-procedural physiological parameters. Therefore, we will attempt to perform intention-to-treat (ITT) analyses wherever possible.

#### Assessment of heterogeneity

We will apply the chi-square test to check for evidence of heterogeneity of intervention effects. In addition to statistical assessments, variability in study participants, interventions and outcomes will be examined qualitatively. We will use the *I*^2^ statistic to describe the percentage of total variation across studies that is due to the heterogeneity rather than chance. An *I*^2^ value greater than 50 % will be considered significant heterogeneity. Visual inspection of the graphical representation of study results with their 95 % CIs will also be used to assess heterogeneity.

#### Assessment of publication bias

We will minimise the risk of publication bias by comprehensively searching databases and a clinical trial registry [14]. We will attempt to obtain data from unpublished work, and no language restriction will be imposed to reduce the risk of reporting bias. We will use a graphical display (funnel plot if greater than ten studies are included) of the size of treatment effect against the precision of the trial to investigate publication bias by looking for signs of asymmetry. If there is asymmetry, we will look for reasons other than publication bias.

#### Data synthesis

The appropriateness of undertaking meta-analysis in the presence of significant clinical or statistical heterogeneity will be considered. If appropriate, we will use the fixed-effect model meta-analysis except where statistical heterogeneity is identified, in which case we will use the random-effects model [12]. RevMan software (version 5) will be used for meta-analyses. Results from individual trials for the outcomes of this review will be tabulated and synthesised in narrative form if meta-analyses cannot be performed because the measure of the outcome is not sufficiently consistent across trials.

#### Subgroup analysis

We will consider conducting subgroup analysis if the data indicates clinical or statistical (*I*^2^ > 50 %) heterogeneity based on the following:

- age (adults or children);
- use of supplemental oxygen (used routinely or not);
- the type of sedation regimen used (propofol, benzodiazepine, benzodiazepine and opioid combination, ketamine, dexmedetomidine, other);
- whether an anaesthetist administered PSA (could have been defined in this instance as monitored anaesthesia care) or not;
- whether pre-specified respiratory depression criteria were provided to guide clinicians’ decision-making; and
- the type of procedure (diagnostic or interventional).

#### Summary of findings

We will present study findings in a standard “summary of findings” (SoF) table, which will include the primary and secondary outcomes, a measure of the typical burden of these outcomes, the absolute and relative magnitude of effect, the numbers of participants and studies addressing each outcome and a grade for the overall quality of the body of evidence for each outcome. We will use the principles of the Grades of Recommendation, Assessment, Development and Evaluation (GRADE) system [15] to assess the quality of the body of evidence associated with the outcomes and will construct the SoF table using GRADE software. The GRADE approach appraises the quality of a body of evidence according to the extent to which one can be confident that an estimate of effect or association reflects the item being assessed. The quality of the body of evidence considers within-study risk of bias (methodological quality), directness of the evidence, heterogeneity of the data, precision of effect estimates and risk of publication bias.

## Discussion

Results from this systematic review will be valuable for clinical practice and research. To the best of our knowledge, this systematic review will examine the current state of evidence on the benefits to patient safety that may be associated with using capnography during PSA in the hospital setting for the first time. Reducing the risk of the most common antecedent event (hypoxia from inadequate oxygenation or ventilation) for sedation-related death and permanent neurological deficits would be a strong indicator that capnography monitoring is likely to improve patient safety during PSA. A synthesis of the existing evidence will assist clinicians to integrate findings into their practice. Gaps in the literature will be identified, which need to be addressed in research to improve patient safety during PSA.

## Additional file

Additional file 1:
**Study eligibility, data extraction form, quality assessment.** The forms found in the file will be used by the reviewers in the course of this systematic review.

## Appendices

### Appendix 1. Search strategy for CENTRAL

#1 MeSH descriptor: [Capnography] explode all trees

#2 Capnograph*ab or end-tidal carbon dioxide:ti,ab

#3 #1 or #2

#4 MeSH descriptor: [Conscious Sedation] explode all trees

#5 MeSH descriptor: [Deep Sedation] explode all trees

#6 #4 or #5

#7 #3 and #6

### Appendix 2. MEDLINE (EBSCOHost) search strategy

1. exp Capnography/ or (capnograph*).mp. or end-tidal carbon dioxide.mp.

2. Conscious Sedation/ or sedat*.ti,ab.

4. 1 and 2

### Appendix 3. CINAHL (EBSCOhost) search strategy

S1 (MH “Capnography”) OR capnograph* or end-tidal carbon dioxide

S2 AB (sedat* OR (MH “Propofol”) OR (MH “Conscious Sedation”))

S4 S1 and S2

## Abbreviations

GRADE: Grades of Recommendation Assessment, Development and Evaluation
ITT: intention to treat
PSA: procedural sedation and analgesia
RevMan: Review Manager
SoF: summary of findings
